# Impact of mood stabilizers on creativity

**DOI:** 10.1177/10398562231211127

**Published:** 2023-10-30

**Authors:** Gordon Parker

**Affiliations:** Discipline of Psychiatry and Mental Health, 7800 University of New South Wales, Sydney, NSW, Australia

**Keywords:** manic depressive illness, bipolar disorder, mood stabilizers, cognitive side-effects

## Abstract

**Objective:**

While a DSM-5 criterion for both hypomania and mania is impaired functioning, the majority of those with a bipolar condition report improved functioning. When offered a mood stabilizer, many express concerns about any impact on their creativity. This piece seeks to address the question and attendant issues.

**Method:**

Reference is made to the impact of differing mood stabilizers on cognitive performance and the limited data on any specific impact on creativity, while some personal observations are offered.

**Results:**

There appears to be a distinctive gradient in the cognitive impacts of differing mood stabilizers, with lithium offering the highest risk, carbamazepine and valproate providing a slight risk, and lamotrigine seemingly without cognitive side-effects.

**Conclusions:**

The question not only invites a nuanced response from the clinician but argues for close observation of any cognitive side-effects when lithium is introduced.

‘One of the biggest reasons why people go off their bipolar medication is they miss that euphoric quick problem-solving, creative dimension that is enhanced by low- to mid-levels of mania’.

*– patient Martin, quoted by Suran.^1^*

‘No pill can help me deal with the problem of not wanting to take pills’.

– *Kay Redfield Jamison.^2^*

A not uncommon question from patients newly diagnosed with a bipolar disorder and having a mood stabilizer recommended is whether the drug will cause them to lose their ‘creativity’.

The query appears particularly cogent for those whose work demands or benefits from creative skills, whether weighted to methodical analytic problem-solving or to ‘aha’ insight expressions, and when it is well documented that bipolar disorder is distinctly over-represented in writers and artists,^3^ as well as in high achievers – including British Prime Ministers.^4^

This paper considers nuances to such a seemingly straightforward question. A review of the literature offers little assistance if an evidence-based answer is sought, reflecting few papers considering the topic and some salient papers being of limited quality. In a clearly dated study, Schou^5^ evaluated subjective effects of lithium in a group of 24 artists and writers who had responded to lithium – with six judging their creativity as unaltered, six as it as having worsened and 12 reporting improvement. Goodwin and Jamison^6^ observed (p 404), however, that Schou’s trial was brief and, in relation to those reporting impaired performance, that there is ‘some indication that patients partially accommodate to lithium’s cognitive effects’.

The frequency of the question argues for considering it from a broader perspective. In pursuing whether a patient might have a bipolar disorder, I ask a dozen or so screening questions about hypo/manic symptoms and find that about 80 per cent of those with a likely bipolar condition affirm feeling ‘more creative’. Supplementary clarifying questions can be informative. A clinical vignette: after affirming the probe question, a final year high school student described how she had been assigned six essays to be completed in 8 weeks. Experiencing a hypomanic episode at that time, she completed all in 3 days and after virtually no sleep received top grades for all essays and with all marks in line with or superior to her base academic level.

There was no doubt that her mood state advanced her ‘creative output,’ but this alone does not mean that her creativity was necessarily enhanced. The high marks were likely to reflect many parameters in play. She had more energy, enthusiasm and some grandiosity of mood, all potential performance enhancers – and with the last aspect not always recognized. For both hypomania and mania, DSM-5 requires a level of impairment. However, an analysis^7^ of data provided by 74 patients with a bipolar I disorder and 104 with a bipolar II disorder established that the majority of those with each condition reported ‘improved functioning’ during hypo/manic states (76% and 86%, respectively).

An additive model may be in play. Johnson et al^8^ undertook a literature review of creative constructs intrinsic to those with a bipolar disorder and reported an over-representation of a number of personality and related traits – including impulsivity, ambition, openness to experience, increased confidence, increased productivity and increased fluency – that may predispose such individuals to greater creative success during hypo/manic episodes.

My impression is that, most commonly, enhanced performance of a latent construct is in play. In support, and also illustrating the interdependence of hypo/manic states and creative performance, is the study by Jamison^9^ of 47 eminent British writers and artists. Virtually, all (89%) had experienced ‘intense, highly productive and creative episodes’. Jamison listed key components of such mood states. In descending order of prevalence, the most common reported changes (and ones that would definitely advance performance and hence creativity) were of enthusiasm, energy, self-confidence, speed of mental association, fluency of thoughts, euphoric mood, ability to concentrate, emotional intensity, sense of well-being, rapid thinking, expansiveness and less need for sleep. Advanced performance is not only evident in creative arenas but in quite differing fields – such as the footballer who can ‘see’ breaks in the opposition’s defence line more rapidly and successfully when hypomanic or the racing car driver who, when hypomanic, reports a greater ability to judge the maximum speed to take a corner.

Thus, the meta-communication underpinning the question is commonly whether the mood stabilizer will compromise the positive experiential and performance-enhancing aspects of the patient’s hypo/manic states – with the clinician’s answer having the potential to influence medication adherence. When the patient is highly distressed and/or disabled by their depressive episodes, most clinicians will effectively bypass the question and simply suggest that if their depressive episodes are to vanish, then they must forgo their highs. Such advice is often accompanied by the clinician pointing out that creativity is almost invariably severely compromised during bipolar depressive episodes.

If a broader answer is to be put, what might be the clinician’s response? I suggest clarifying whether the question is it in reference to a loss of creativity associated alone with a hypo/manic episode or refers to their intrinsic creativity. The former allows that during a hypo/manic episode, an individual may become more creative, and if their bipolar disorder is brought under control, then it must be expected that, no longer touched by fire, they must be expected to lose that value-added component. The latter allows that the individual is intrinsically highly creative and that mood stabilizing medication might significantly compromise their performance even when euthymic.

For both scenarios, the answer depends on the choice of mood stabilizer. The literature on the degree to which differing mood stabilizers have differing propensities for compromising creativity is sparse but seemingly clear cut if we consider the risk of cognitive disturbance with lithium providing the greatest risk. Polatin and Fieve^10^ reported (p 864) that lithium was rated negatively by creative individuals, with lithium acting as a ‘brake’, lessening drive, incentive and ability to express themselves. In such instances, lithium compromised ‘performance’ initially and any creativity component downstream.

Goodwin and Jamison^6^ noted that there have been no controlled studies of lithium’s effects on productivity in creative people and then overviewed several uncontrolled studies, with conflicting findings possibly reflecting participants in those studies variably having a bipolar disorder (and where detrimental effects were reported) or being ‘normal’ subjects (and where such effects were not evident). If lithium does risk compromising creativity, it also remains unclear whether such a side-effect is dependent on the serum level.

My impression is that lithium is more likely to cause cognitive side-effects in those with a bipolar II as against a bipolar I condition, and with there being high rate in those with a bipolar II condition, and that cognitive compromising can occur even when serum lithium levels are low or sub-threshold. In a randomized controlled 20-week trial^11^ comparing lithium and lamotrigine as maintenance treatments in those with a bipolar II condition, 50% of trial completers had severe cognitive side-effects, as reflected in them reporting cognitive slowing, impaired memory and/or word-finding difficulties despite their lithium levels being in the study range of 0.6–1.0 mEq/l.

For creative individuals, a move from lithium to another mood stabilizer in such circumstances is clearly worthy of consideration. There is seemingly only one salient study. Stoll et al^12^ reported that valproate was more protective of creativity than lithium in reducing ‘the cognitive, motivational or creative deficits attributed to lithium’; however, their sample involved only seven subjects.

There appear to be no studies evaluating the impact of other mood stabilizers on creativity and so the issue has to be addressed indirectly – by simply considering the extent to which the differing mood stabilizers induce cognitive side-effects. Eddy et al^13^ undertook an embracive review of antiepileptic drugs on cognitive function and reported that carbamazepine had detrimental effects on memory and verbal fluency, there being several studies indicating little effect of valproate on cognition and other studies indicating no effect of lamotrigine on cognition. A gradient is seemingly evident with lithium providing the greatest risk and then progressively lower risks being presented by carbamazepine, valproate and then lamotrigine. In addition, there is some anecdotal data that lamotrigine can slightly increase creativity, in particular when it is taken at higher doses (of 250 mg or more) with Woollacott et al^14^ reporting the case of a woman with epilepsy commencing to write 10–15 poems a day after commencing lamotrigine. Compromised creativity may lead to cessation of medication, but such a risk may occasionally be averted by the use of augmenting medications such as stimulant drugs (for bipolar depressive episodes) which may reduce any compromising of creativity by the mood stabilizer.

## Conclusion

Thus, in response to ‘the question’, if the recommended mood stabilizer is lamotrigine, then the patient can be reassured. If it is carbamazepine or valproate, then a low risk answer can be provided. If lithium is the recommended mood stabilizer, then the risk of compromised cognitive performance is real and may be more likely in those with a bipolar II than a bipolar I disorder. The question therefore benefits from a nuanced clinician response and serves as a signal component to be assessed carefully when a mood stabilizer is initiated.
